# Significantly enhanced osteoblast response to nano-grained pure tantalum

**DOI:** 10.1038/srep40868

**Published:** 2017-01-13

**Authors:** W. T. Huo, L. Z. Zhao, S. Yu, Z. T. Yu, P. X. Zhang, Y. S. Zhang

**Affiliations:** 1 Northwest Institute for Nonferrous Metal Research, Xi’an 710016, China; 2 State key Laboratory of Military Stomatology, Department of Periodontology, School of Stomatology, The Fourth Military Medical University, Xi’an 710032, China

## Abstract

Tantalum (Ta) metal is receiving increasing interest as biomaterial for load-bearing orthopedic applications and the synthetic properties of Ta can be tailored by altering its grain structures. This study evaluates the capability of sliding friction treatment (SFT) technique to modulate the comprehensive performances of pure Ta. Specifically, novel nanocrystalline (NC) surface with extremely small grains (average grain size of ≤20 nm) was fabricated on conventional coarse-grained (CG) Ta by SFT. It shows that NC surface possessed higher surface hydrophilicity and enhanced corrosion resistance than CG surface. Additionally, the NC surface adsorbed a notably higher percentage of protein as compared to CG surface. The *in vitro* results indicated that in the initial culture stages (up to 24 h), the NC surface exhibited considerably enhanced osteoblast adherence and spreading, consistent with demonstrated superior hydrophilicity on NC surface. Furthermore, within the 14 days culture period, NC Ta surface exhibited a remarkable enhancement in osteoblast cell proliferation, maturation and mineralization as compared to CG surface. Ultimately, the improved osteoblast functions together with the good mechanical and anti-corrosion properties render the SFT-processed Ta a promising alternative for the load-bearing bone implant applications.

Recently tantalum (Ta) metal is receiving increasing interest as biomaterial for load-bearing orthopedic applications due to its excellent biocompatibility (*e.g.*, outstanding bone-like apatite forming capability in simulated body fluid (SBF), no cytotoxic ion release or dissolution in local, systemic and remote organs, as well as good osseointegration), superior strength, as well as anti-corrosion properties^1^,^2^,^3^,^4^,^5^. Particularly, *in vitro* studies^3^,^6^ comparing the Ta implant and the common titanium (Ti) implant show that the bioactivity and cell-material interactions are significantly better in the case of Ta, further indicating Ta exhibits high promise for bone implant application.

As we know, substrate grain structure and topography of the implant materials plays a crucial role in mediating cell-substrate interactions at the implant/tissue interface, and microstructure modification such as grain refinement is frequently used to tailor the physical and biological properties of various biomaterials. For example, fine-grained (in sub-microscale or nanoscale) structures exhibiting significantly enhanced mechanical properties have been successfully fabricated in 316 L stainless steel^7^,^8^, pure Ti and its alloys^9^,^10^,^11^,^12^,^13^, as well as zirconium (Zr)^14^, more importantly, a remarkable enhancement in corrosion resistance and cellular activity have also been observed on those fine-grained surfaces, in comparison with their coarse-grained counterparts. With regard to Ta, due to the high plastic deformation resistance of typical body-centered cubic (BCC) metal, nanocrystalline (NC) Ta is generally hard to obtain and accordingly the biological performance of NC Ta has scarcely been explored to our knowledge.

Surface mechanical grinding treatment (SMGT)^15^,^16^ or sliding friction treatment (SFT)^17^,^18^,^19^ with quite high strain rate of about 10^3^–10^4^ s^−1^ is a favorable severe plastic deformation (SPD) method that can generate a solid layer of nanocrystalline structure on the metal surface. Recently, NC Ta surface with an average grain size of ≤20 nm has been successfully manufactured through the SFT technique developed by us^17^,^18^,^19^. The detailed microstructural evolution^17^, corresponding grain-refinement mechanisms, as well as advantages in mechanical properties^19^ have also been explored systematically. It is of great interest to explore the potential of this NC Ta surface for bone implant application. Therefore, in this work, we investigated the *in vitro* bioactivity of the novel NC Ta surface (Fig. 1b). The conventional coarse-grained (CG) Ta (Fig. 1a) without the SFT treatment was set as control.

## Results

### Grain structure

**Figure 1:**
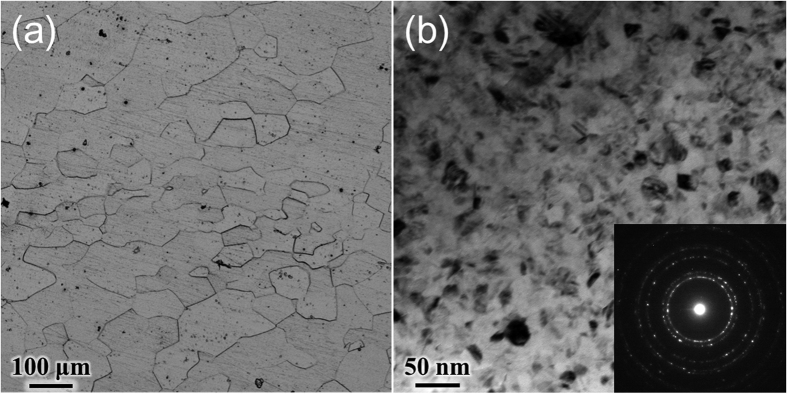
Grain structures of the (**a**) CG Ta and (**b**) NC Ta samples.

The grain structures of Ta samples before and after the SFT treatment are presented in Fig. 1. The initial CG Ta sample owns an equiaxed grain structure with an average grain size of ≥60 μm (Fig. 1a). Owing to the much higher strains and strain rates induced by SFT, a thicker (thickness: ~280 μm) process-influenced layer was generated on Ta surface^17^, and extremely small grains with an average grain size of ≤20 nm were obtained in the topmost 20 μm-thick surface layer of the NC samples (Fig. 1b). Moreover, the effective grain refinement on SFTed Ta surface could also be confirmed by the X-ray diffraction (XRD) results in ref. 17, which revealed that NC Ta sample possessed notably broader Bragg peaks as compared to the CG Ta matrix.

### Surface roughness and contact angle

**Figure 2:**
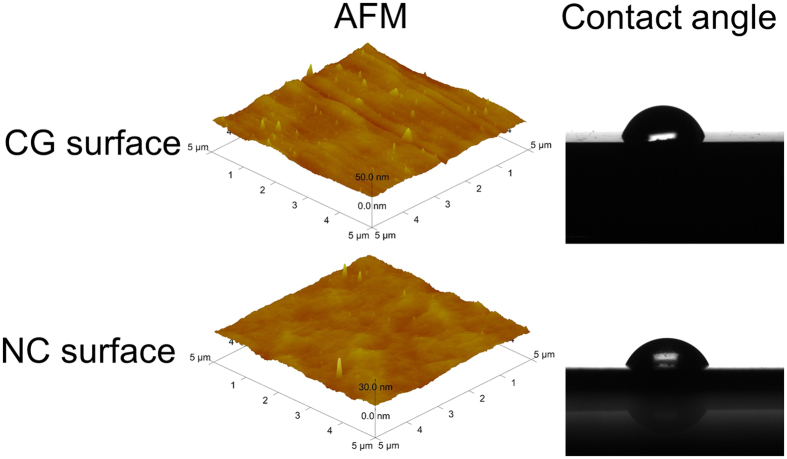
AFM images and water contact angles of the CG and NC Ta surfaces.

Figure 2 shows that the mirror polished CG and NC Ta surfaces display similar roughness and topography as imaged by atomic force microscopy (AFM). The mean values ± standard deviation (SD) of the roughness of the samples in a 5 × 5 μm^2^ area were 1.87 ± 0.21 nm for the CG Ta surface and 1.04 ± 0.13 nm for the NC Ta surface. There was no significant difference in the roughness between the CG and NC Ta samples. The contact angles of the water droplets on the CG and NC Ta surfaces were 67.3 ± 3.1° and 59.4 ± 1.6°, respectively, as shown in Fig. 2, indicating that the NC Ta surface exhibited higher wettability than the CG Ta surface. The increased hydrophilicity on the NC Ta surface may derive from the significantly increased grain boundaries, as well as other factors such as improved atom activity and air adsorption capacity induced by the nanoscaled surface^20^,^21^,^22^,^23^.

### Electrochemical tests

**Figure 3:**
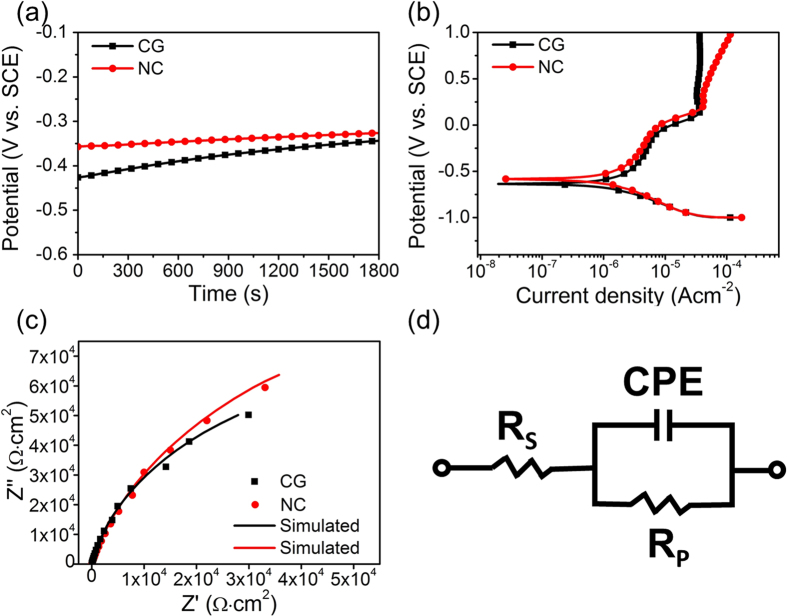
Electrochemical measurements of the CG and NC Ta samples. (**a**) OCP curves, (**b**) polarization curves, (**c**) Nyquist plots from electrochemical impedance spectroscopy (EIS), (**d**) equivalent circuit for analysis of the EIS spectra.

The changes in open circuit potential (OCP) of NC and CG Ta samples measured as a function of time are shown in Fig. 3a. Figure 3b exhibits the potentiodynamic polarization (PDP) curves of these samples and the corrosion potential (*E*_*corr*_) and corrosion current density (*I*_*corr*_) are summarized in Table 1. The OCP of the NC Ta sample shifted towards the more positive direction compared to that of the CG Ta sample (Fig. 3a), indicating that a more stable film may be favorably formed on the NC Ta surface^4^,^24^,^25^. Similarly, the *E*_*corr*_ for the NC Ta sample stayed higher than that for the CG Ta sample (Table 1). It is well-documented that the more positive the *E*_*corr*_ is, the nobler the material is^26^,^27^,^28^. Therefore, the result of PDP curves also implies that the corrosion resistance of NC Ta is enhanced by reducing the grain size from microscale to the nanoscale.

In order to evaluate the effect of grain refinement on the structure of the passive films formed on NC and CG Ta substrates, Nyquist plots drawn from the electrochemical impedance spectroscopy (EIS) spectra were also analyzed. Figure 3c demonstrates that the Nyquist plots were characterized by single semicircles, suggesting the involvement of single time constant. Thus, a single time constant model as shown in Fig. 3d was used to fit EIS results, and the specific values are presented in Table 1. In Fig. 3d, *R*_*s*_ and *R*_*p*_ represent solution and passive film resistance, respectively; *CPE* is the capacitance of the passive film represented by the constant phase element. Figure 3c corroborates that the fitted data derived from the equivalent circuit model agrees well with the experimental data, with deviation less than 3%. Obviously, the results reveal the passive film formed on the NC Ta sample exhibits higher resistance (with higher *R*_*P*_ values) than that on CG sample in SBF solution. With regard to the aforementioned *CPE*, its impedance can be calculated in accordance with the Eq. (1)^24^,^29^,^30^:


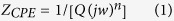


where *w* is an angular frequency (*w* = 2πf), *j* is an imaginary unit, *n* is the exponent of *CPE* ranging from −1 to 1, and *Q* is the capacitance value of the passive film. As shown in Table 1, the values of *n* are close to 1 for NC and CG Ta samples, indicating that the passive films formed on both samples are near ideal capacitors with compact structures. In addition, the capacitance *Q* is associated with the nature of the passive film and can be defined as^24^:


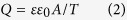


where *ε* is the dielectric constant of passive film, *ε*_0_ is the dielectric constant of free space, *A* is the surface area of working electrode and *T* is the thickness of passive film. Eq. (2) reveals that the higher *Q* corresponds to a lower thickness of the passive film. Herein, the fitted *Q* values listed in Table 1 indicate that the passive film formed on NC Ta sample was thicker than that on CG sample, which is consistent with the result of OCP in Fig. 3a. Since the passive film formed on surface can provide protection for metals, the thicker passive film formed on NC Ta sample would be more beneficial to prevent the metal from corrosion. Hence, the results of electrochemical tests including OCP, PDP, as well as EIS systematically corroborate that the corrosion resistance of NC Ta sample is significantly enhanced than that of CG Ta, which is in line with the previous discovery on the superior anti-corrosion performance for most of the fine-grained materials (*e.g.*, ultra-fine grained Ti, Cu and Fe)^24^,^31^,^32^,^33^,^34^.

### Protein adsorption

**Figure 4:**
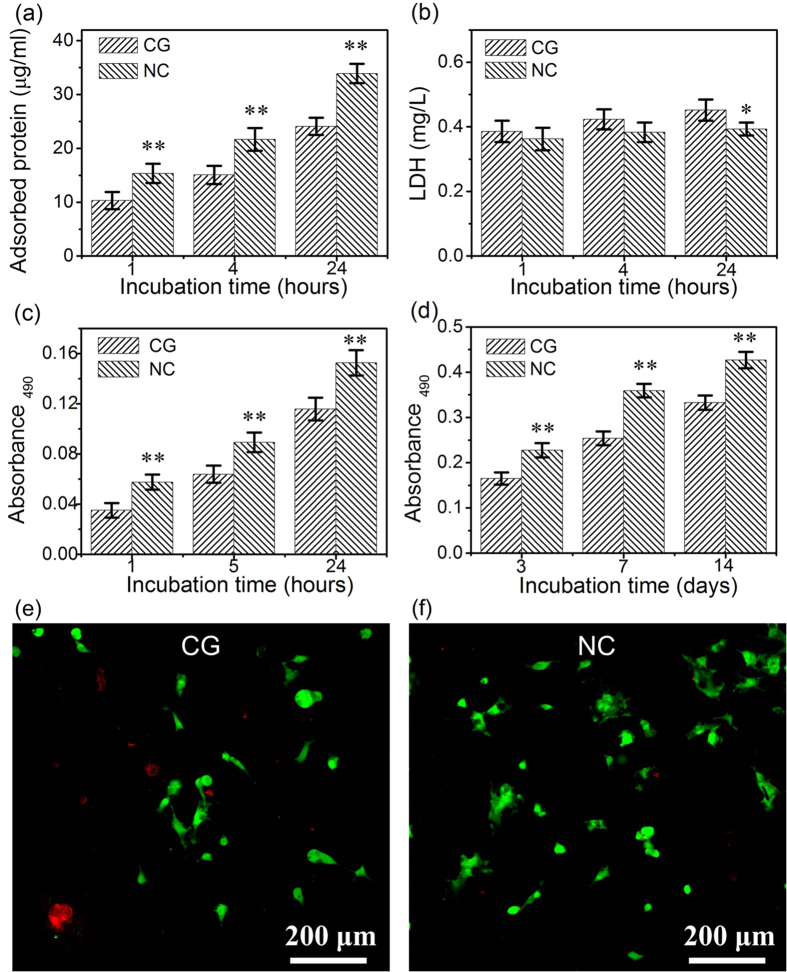
(**a**) Protein adsorption onto the Ta samples after 1, 4 and 24 h of incubation in the FBS-containing DMEM medium, (**b**) cytotoxicity assay by evaluating the LDH activity in the cell culture medium, (**c**) cell adhesion measured by the MTT assay, (**d**) proliferation measured by the MTT assay. (**e**,**f**) Cells incubated for 3 days on (**e**) CG and (**f**) NC Ta samples stained with two well-described probes, indicating live cells (green) and dead ones (red). Data are presented as the mean ± SD, n = 4, *p < 0.05 and **p < 0.01 compared with the CG Ta surface.

The osteoblast is anchorage-dependent cells and the protein adsorption onto the biomaterial surface is the initial critical step that determines the normal cell functions such as cell adhesion and spreading. The amounts of total protein adsorbed from the serum containing Dulbecco’s modified eagle’s medium (DMEM) on the Ta surfaces after 1, 4 and 24 h of incubation are presented in Fig. 4a. With increasing incubation time, the adsorption amounts of total protein tended to increase continuously. At each incubation time point, the NC Ta surface significantly promoted the protein adsorption compared to the CG Ta surface.

### Cytotoxicity

The cytotoxicity indicated by the lactate dehydrogenase (LDH) activity in the culture medium after 1, 4 and 24 h of incubation was assayed (Fig. 4b). The NC Ta surface showed similar and even slightly lower cytotoxicity compared to the CG Ta surface, displaying good cytocompatibility.

### Cell adhesion and proliferation

The human fetal osteoblast (hFOB1.19) cells showed significantly better attachment on the NC Ta surface than the CG surface, even after the first hour of culture (Fig. 4c), which is consistent with the enhanced protein adsorption on the NC Ta surface (Fig. 4a). Figure 4d illustrates the proliferation of hFOB1.19 cells with prolonged incubation time to 14 days. For all culture durations, hFOB1.19 cells proliferated in greater numbers on the NC Ta surface compared to the CG Ta surface. Particularly, after 3 days culturing, the cell proliferation on the NC Ta surface was already notably higher than that on CG Ta surface, which could also be supported by the fluorescent cell viability staining images in Fig. 4e and f (much more live cells and less dead ones were observed on NC Ta surface than the CG Ta surface).

### Cell morphology

**Figure 5:**
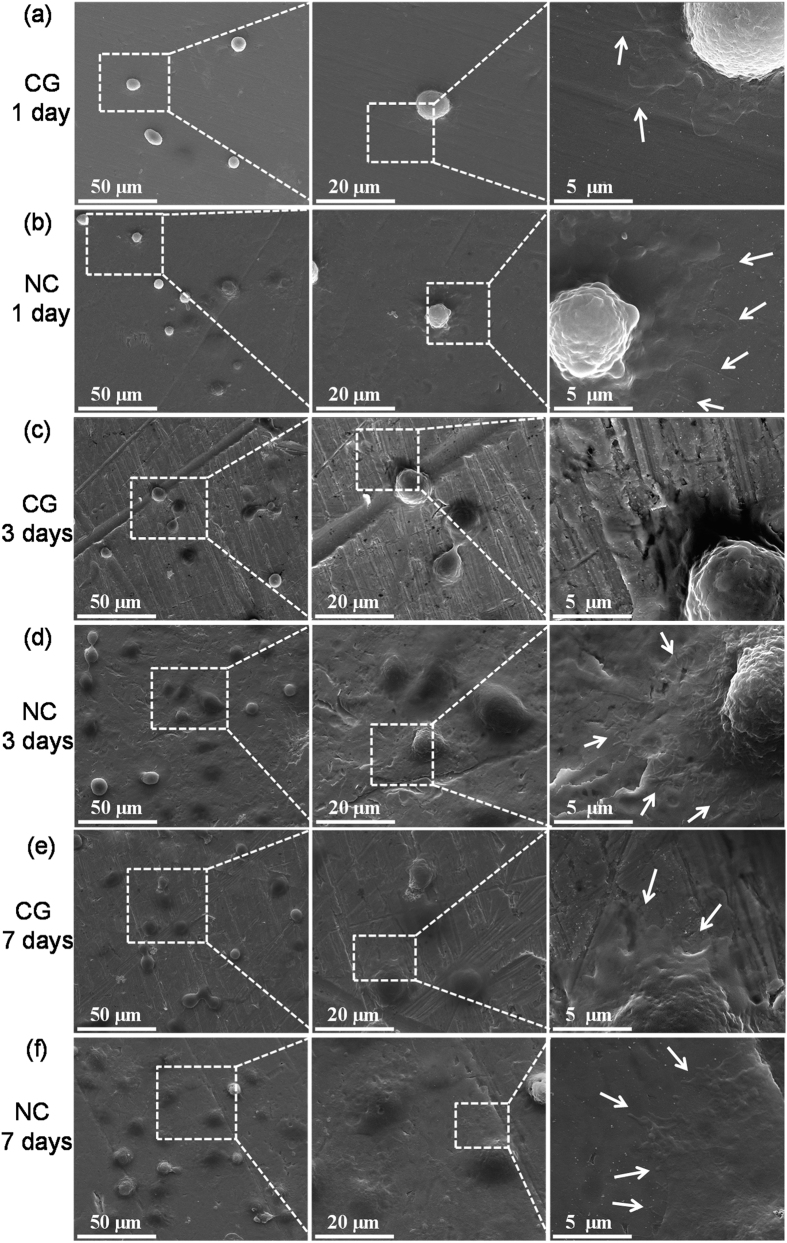
The typical morphologies of the cells cultured on the (**a**,**c**,**e**) CG and (**b**,**d**,**f**) NC Ta surfaces for (**a**,**b**) 1 day, (**c**,**d**) 3 days and (**e**,**f**) 7 days. Arrows indicate filopodia extensions.

The cell shape on a biomaterial surface is closely related to the cell functions. In order to observe cell adhesion and spreading, hFOB1.19 cells cultured on different Ta surfaces were examined by field emission scanning electron microscope (FESEM) after 1, 3 and 7 days of culture (Fig. 5). The better osteoblast attachment on the NC Ta surface was visible within the first day of culture (Fig. 5b). After day 3 and 7, the cell density on NC Ta surface was significantly higher than that on the CG Ta surface (Fig. 5c–f). Additionally, osteoblast appeared to strongly adhere to the surface of NC Ta, supported by the presence of extensive filopodia (indicated by arrows in Fig. 5), flattened morphology, and excellent spreading in multi-directions. These features were less pronounced on the CG surface, further indicating the superior cytocompatibility of the NC Ta surface.

### Osteogenesis-related gene expressions

**Figure 6:**
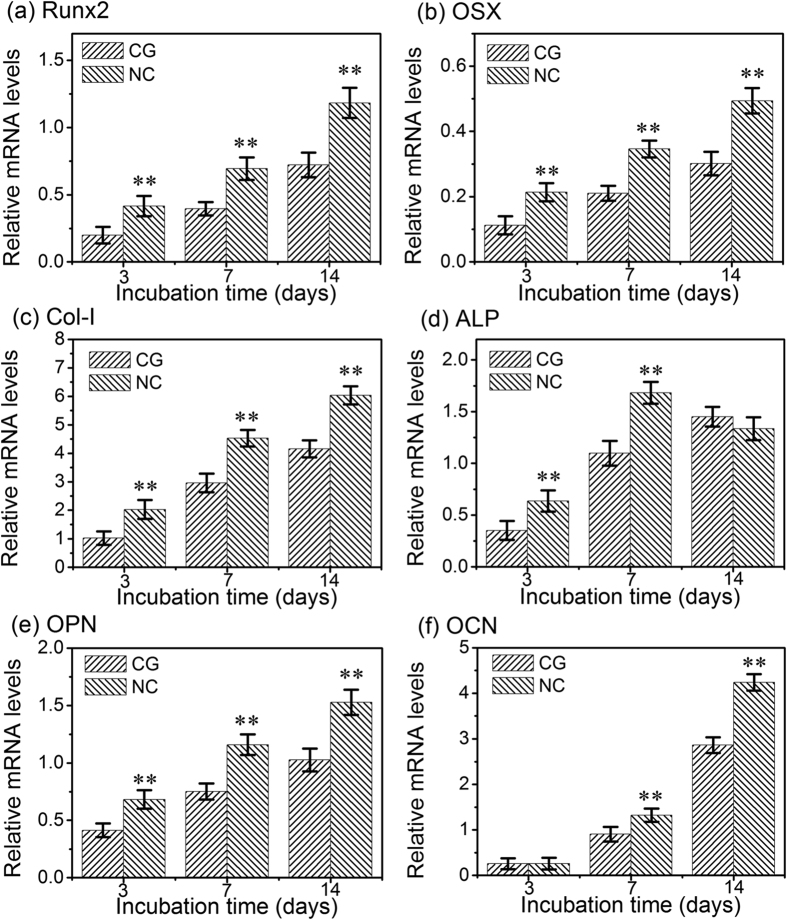
The gene expressions of osteoblasts cultured on the CG and NC Ta surfaces of 3, 7 and 14 days: (**a**) Runx2, (**b**) OSX, (**c**) Col-I, (**d**) ALP, (**e**) OPN and (**f**) OCN. Data are presented as the mean ± SD, n = 4, **p < 0.01 compared with the CG surface.

The hFOB1.19 cells’ differentiation on the CG and NC Ta surfaces can be monitored via measuring the expressions of osteogenesis-related genes. The expressions of runt related transcription factor 2 (Runx2), osterix (OSX), alkaline phosphatase (ALP), osteopontin (OPN), osteocalcin (OCN) and collagen I (Col-I) in cells cultured for 3, 7 and 14 days are shown in Fig. 6. Except for ALP, the mRNA expressions of Runx2, OSX, Col-I, OPN and OCN in the cells cultured on both Ta surfaces increased with incubation time from 3 to 14 days. Besides, the expressions of all the six genes by the cells were significantly higher on the NC Ta surface compared to those on the CG Ta surface at nearly all incubation time points, except that the expressions of ALP and OCN showed nearly no difference among the two Ta surfaces at 14 and 3 days, respectively.

### Intracellular ALP activity, OPN, OCN and Col-I contents

**Figure 7:**
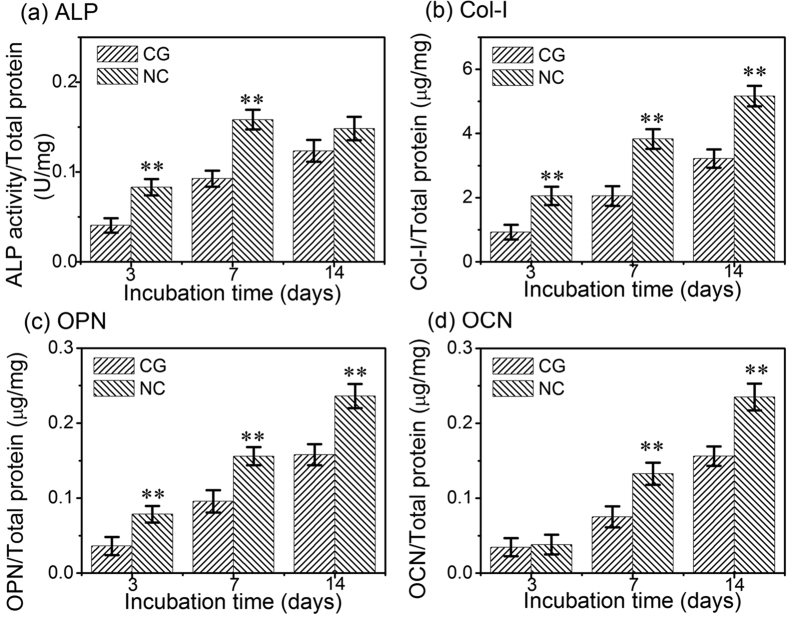
(**a**) The ALP activity and the contents of (**b**) Col-I, (**c**) OPN and (**d**) OCN proteins in osteoblasts cultured on the CG and NG Ta surfaces for 3, 7 and 14 days. Data are presented as the mean ± SD, n = 4, **p < 0.01 compared with the CG Ta surface.

The intracellular ALP activity and the OPN, OCN and Col-I contents in the cells cultured on the CG and NC Ta surfaces for 3, 7 and 14 days are shown in Fig. 7. Overall, the values of these parameters increased with incubation time and the NC Ta surface induced significantly higher ones compared to the CG Ta surface, except that the ALP activity and the OCN content showed nearly no obvious difference among the two Ta surfaces at 14 and 3 days, respectively.

### Collagen secretion and extracellular matrix (ECM) mineralization

**Figure 8:**
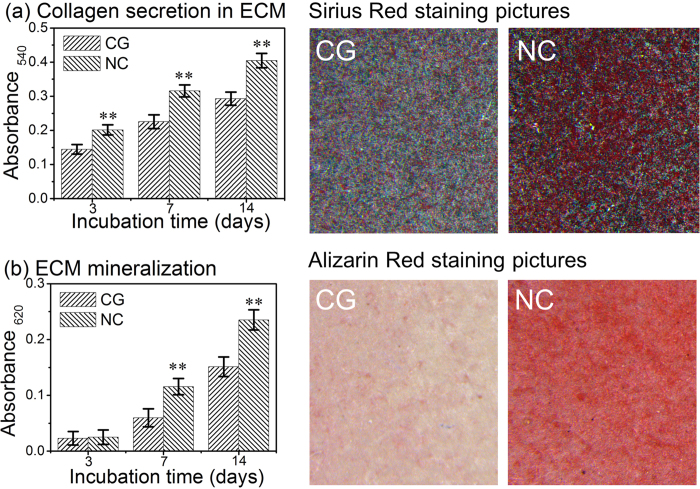
(**a**) The ECM collagen secretion determined with the Sirius Red staining, and (**b**) the ECM mineralization determined with the Alizarin Red staining after 3, 7 and 14 days of osteoblasts incubation on the Ta surfaces. The staining pictures of collagen secretion on the right side of (**a**) were taken after 7 days of incubation, and the staining pictures of ECM mineralization on the right side of (**b**) were taken at 14 days. Data are presented as the mean ± SD, n = 4, **p < 0.01 compared with the CG surface.

The ECM collagen secretion and mineralization by osteoblasts on the CG and NC Ta surfaces after 3, 7 and 14 days of incubation, determined with the Sirius Red and Alizarin Red staining, respectively, are shown in Fig. 8. The NC Ta surface dramatically promoted the collagen secretion and ECM mineralization compared to the CG Ta surface.

## Discussion

In terms of implant materials to be used as substitutes of high load-bearing bones, it is a challenge to explore and design materials that own both favorable surface and bulk properties to regulate the cell-substrate interactions and guarantee the long-term stability. The advantages in the mechanical properties of SFT treated Ta were investigated in previous study, which showed that the yield strength values of Ta had been improved from 300 MPa to 720 MPa after the SFT treatment^19^. Additionally, the NC Ta surface exhibited much higher hardness (Hv = 270) than the CG matrix (Hv = 115), which will provide better wear resistance and decrease the occurrence of metallic debris during high-load functioning^35^. Our present study demonstrated that the NC Ta surface also possessed superior anti-corrosion property as compared to the CG Ta sample (Fig. 3, Table 1), this helps to create a more stable microenvironment that benefits the cellular functions at the cell-substrate interface as well^36^. Excitingly, the NC Ta surface induced more protein adsorption and enhanced osteoblast adhesion, proliferation and differentiation compared to the CG Ta surface (Figs 4–8).

As aforementioned, the protein adsorption onto the biomaterial surface is the initial key step that determines the cell/biomaterial interaction, because the cells sense the foreign surface through the adsorbed protein layer. It has been generally accepted that the protein adsorption and cell response to the substrate can be altered by the physico-chemical properties of the substrate^37^,^38^. The adsorption of protein depends strongly on these factors including surface roughness, chemical composition, ionic binding, grain structure and hydrophilicity^39^,^40^. In previous works, we have shown through the X-ray photoelectron spectroscopy analysis that the NC Ta surface is similar in composition to the untreated CG Ta surface^19^. Additionally, the surface roughness was also similar between the CG and NC Ta surfaces (Fig. 2). Therefore, the hydrophilicity and grain size are the relevant factors contributing to the difference in the protein adsorption amount and cell response. The NC Ta surface demonstrated lower water contact angle compared to the CG Ta surface (Fig. 2), indicating that the NC Ta surface is more hydrophilic and has a higher surface energy than the CG one. The hydrophilic surface with higher surface energy was reported to enhance the adsorption of more anchoring proteins such as fibronectin and vinculin, which promoted a favorable osteogenic microenvironment^11^. Furthermore, it was claimed that the increased grain boundaries provided optimal sites for osteoblast adhesion and by this way the ultrafine-structured surface could improve the cell behaviors^10^. Herein, the extremely increased grain boundaries of the NC Ta surface may provide more optimal sites for focal adhesion of cells, which shall be another reason for the enhanced osteoblast response. Thus, the increased surface wettability and the presence of nanocrystalline structure shall contribute to the notably improved protein adsorption and subsequently significantly pronounced cell adhesion on the NC Ta surface as compared to the CG Ta surface. This is in agreement with a large number of reports demonstrating that the ultra-fine structured surfaces led to enhanced protein adsorption and cell spreading^7^,^9^,^10^,^11^,^23^,^33^,^41^,^42^,^43^.

Higher cell proliferation can result in more cell colonization accompanied with more ECM secretion on the implant surface probably leading to a larger mass of bone tissue around the implant. In present work, the proliferation of hFOB1.19 cells was enhanced on the NC Ta surface compared to the CG Ta surface, which is in good agreement with the cell adhesion result. The osteoblast proliferation is also marked by the induction of ECM composed of collagen and non-collagenous proteins. The detected collagen in Fig. 8a at day 3 indicated that the ECM formed relatively earlier on the NC Ta surface and its content was considerably more than those on the CG Ta surface, indicating again that the cell proliferation as well as ECM synthesis were accelerated on the NC Ta surface, probably inducing faster bone maturation around the implant.

To achieve optimal bone healing around the implant, the subsequent osteoblast functions related to differentiation are also required. The differentiation process of hFOB1.19 cells on the two kinds of Ta surfaces was evaluated by the mRNA expressions of osteogenesis related genes (including ALP, Runx2, OSX, OPN, OCN and Col-I), ALP activity, as well as the OPN, OCN and Col-I protein product. The events occurring in a chronological order during the osteoblast differentiation include: firstly the enhancement of ALP activity, then synthesis of Col-I, and finally deposition of the non-collagenous ECM proteins, such as OPN and OCN to form mineralized bone^37^. ALP is regarded as an early marker of osteoblast differentiation, with peak mRNA and activity levels pronouncing osteoblast maturation, and the expression level then decreases at the onset of mineralization^11^,^36^. Our results demonstrated higher mRNA level and activity of ALP on the NC Ta surface compared to the CG Ta surface at 3 and 7 days (Figs 6d and 7a), indicating that the osteoblast cultured on the NC Ta surface differentiated to a more mature phenotype. Moreover, both the mRNA level and activity of ALP on the NC Ta surface decreased when the incubation period exceeded 7 days, further suggesting that the cell differentiation were accelerated on this surface, which progressed into the ECM mineralization phase at day 7. Runx2 is a transcription factor necessary for bone formation^44^, and OSX is a zinc finger transcription factor specifically expressed by osteoblasts to induce the differentiation of themselves to the mature phenotype^45^. The higher expression mRNA levels of Runx2 and OSX on the NC Ta surface (Fig. 6a and b) compared to the CG one further demonstrated that the cell differentiation was accelerated by NC Ta surface by means of promoting a mature phenotype at earlier stage. The enhanced expression of OPN, OCN and Col-I at both gene and protein levels by the NC Ta surface (Figs 6 and 7) also corroborated this. These osteogenic proteins are directly regulated by Runx2 and OSX through their DNA binding element^46^. Col-I is the main ECM protein in bone and one of the most widely recognized biochemical markers in osteoblast differentiation^47^,^48^. OPN, whose expression increases both in early differentiation and during mineralization, is known to be a middle-stage maker and be associated with the onset of ECM mineralization at a later point^10^,^49^,^50^. OCN, being capable of binding ECM calcium to the developing bone matrix, is expressed at the later stages of bone matrix formation^51^ and the ECM deposition is closely regulated by OCN^47^. It is found here that the NC Ta surface remarkably out-performed the CG Ta surface in inducing higher mRNA expression of Col-I, OPN and OCN (Fig. 6), promoting their intracellular protein production (Fig. 7), expediting the collagen secretion (Fig. 8a), and accelerating the ECM mineralization (Fig. 8b). These results collectively certify that the NC Ta surface can accelerate the differentiation of hFOB1.19 osteoblast, resulting in improved cell maturation and enhanced ECM mineralization.

## Methods

### Sample preparation

The details of the SFT set-up and the sequences of the SFT processing of pure (99.95 wt%) Ta were given in ref. 19. The given processing parameters for this study were as follows: 500 N in load, 0.2 m/s in sliding velocity, 100 μm in offset displacement perpendicular to the sliding direction after each sliding direction, and 100 in cycle. Square samples of 10 × 10 mm^2^ cut from both the SFT treated and untreated Ta sheets were ultrasonically cleaned in acetone, ethanol, and distilled water for 10 min each and dried in air. For *in vitro* biocompatibility studies, all samples were sterilized with alcohol for 3 h and rinsed twice with sterile phosphate buffer saline (PBS; Sigma, USA) before cell seeding.

### Microstructure characterization

The surface microstructure of the NC Ta sample was characterized by a JEOL JEM-2100 transmission electron microscope (TEM) operated at 200 kV. The TEM samples were cut from the top surface layer and thinned by ion thinning at low temperature. The surface grain structures of the CG Ta sample were observed by Olympus PMG 3 optical microscopy (OM).

### Electrochemical tests

Electrochemical measurements were carried out using an IM6 Zahner-electrik Gmbh (Zenniom, Germany) electrochemical workstation. A standard three-electrode setup was used to perform electrochemical measurement: *e.g.*, a test sample, a platinum wire and a saturated calomel electrode (SCE) act as working electrode, counter electrode and reference electrode, respectively. OCP, PDP curve and EIS tests were all carried out on in SBF solution (prepared according to ref. [52]) at temperature of 37 °C. The OCP measurement was set for 1800 s. The PDP curves were carried out from −1.0 V (vs. SCE) to +1.0 V (vs. SCE) at a scanning rate of 0.01 V/s. The EIS measurements were carried out over a frequency range from 100 kHz to 10 mHz with a 10 mV amplitude AC voltage signal. Nyquist plots were obtained after the samples were immersed in the solution for 1 h to evaluate the characteristics of passive films formed on the surface of Ta, and the EIS parameters were determined from the Nyquist plots after fitting the data using ZSimpWin 3.0 software.

### Surface roughness and wettability

The surface roughness was recorded using AFM (Dimension ICOM, America) scanning with triplicated repeats. To characterize how the fine-grained structure changes the surface properties of Ta, the surface wettability test was performed using a Kino SL200B contact angle system.

### Protein adsorption assay

For the protein adsorption assay, a 500 μL droplet of DMEM/Ham’s F12 1:1 medium (Thermo Scientific, USA) containing 10% fetal bovine serum (FBS; Thermo Scientific, USA) was pipetted onto each sample placed in a 48-well plate. After incubation at 37 °C for 1, 4 and 24 h, these samples were transferred to a new 48-well plate and washed with 500 μL PBS thrice. Afterwards, 250 μL of 1% sodium dodecyl sulfate (SDS) solution was added to these wells and shaken for 15 min to detach proteins from the Ta surfaces. The protein concentrations in the collected SDS solutions were determined using a MicroBCA protein assay kit (Pierce, USA). The total amounts of protein adsorbed on the Ta surfaces were determined using a standard curve drawn with BSA. Four samples of each group were tested, and each test was repeated four times (n = 4).

### Cell culture

The hFOB1.19 cells (provided by the Institute of Biochemistry and Cell Biology of Chinese Academy of Sciences, Shanghai, China) were cultured in DMEM supplemented with 10% FBS, 0.3 mg/mL Geneticine418 (Sigma, USA), 0.5 mM sodium-pyruvate (Sigma, USA) and 1.2 mg/L Na_2_CO_3_ at 37 °C in a humidified atmosphere of 5% CO_2_. The medium was refreshed every 2 days during cell culturing.

### Cell adhesion and proliferation

The cell adhesion and proliferation were determined employing the MTT (3-(4,5-dimethylthiazol-2-yl)-2,5-diphenyltetrazolium bromide) assay. The cells were seeded on the Ta samples (four replicates) in 24-well plates at a density of 8 × 10^4^ cells/well. After cultured for 1, 5, 24, 72, 168 and 336 h, the culture medium was discarded, and the samples were washed thrice with PBS then incubated at 37 °C for another 4 h in fresh culture medium containing MTT solution (0.5 mg/mL medium). After the unreacted dye was removed, the intracellular purple formazan product was dissolved into a colored solution by adding the dimethyl sulfoxide to the wells. The optical density (OD) was then measured at 490 nm using a spectrophotometer. Each test was repeated four times (n = 4).

### Cytotoxicity assay

The LDH activity in the culture medium was used as an index of cytotoxicity. After 1, 4 and 24 h culturing, the culture medium was collected and centrifuged, and the supernatant was used for the LDH activity assay. The LDH activity was determined spectrophotometrically according to the manufacturer’s instructions.

### Cell morphology

After 24, 72 and 168 h culturing, the cell adherent samples were washed three times with PBS, and fixed with 2.5% glutaraldehyde for 1 h at 4 °C. The fixed samples were then dehydrated in ethanol, followed by vacuum drying. After gold-palladium coating, the cell morphology was observed under FESEM (JSM-6700F, Japan).

Live/dead staining using the LIVE/DEAD Viability/Cytotoxicity Kit (Invitrogen, France) was performed to identify viable and nonviable hFOB1.19 on the samples after 3 days of incubation. The cell-adherent samples were incubated with 500 μL of PBS containing ethidium-homodimer-1 (4 μM) and calcein-AM (2 μM) at 37 °C for 30 min. The fluorescence-stained cells were analyzed using an OLYMPUS laser confocal microscope (FV1000).

### Osteogenesis-related gene expressions

After culturing for 3, 7 and 14 days, the total RNA was isolated using the TRIzol reagent (Gibco, USA). RNA (1 μg) from the cells on each sample was reverse transcribed into complementary DNA using the PrimeScrip™ RT reagent kit (TaKaRa, Japan). The expressions of Runx2, OSX, ALP, OPN, OCN and Col-I were quantified using a real-time polymerase chain reaction (qRT-PCR) detection system (Bio-Rad iQ™5 Multicolor) with SYBR® Premix Ex™ Taq II (TaKaRa, Japan). The data analysis was carried out using a iQ™5 Optical System (Bio-Rad, USA) with Software Version 2.0. The expression levels of the target genes were normalized to that of the housekeeping gene glyceraldehyde-3-phosphate dehydrogenase (GAPDH). The sequences of the specific primer sets are listed in Table 2. Four samples from each group were tested, and each test was repeated four times (n = 4).

### Intracellular ALP activity, OPN, OCN and Col-I contents

The cell-seeded samples were washed thrice with PBS, and then lysed in 0.1 vol.% Triton X-100 through five standard freeze-thaw cycles and shaken for 10 min. The intracellular ALP activity and contents of OPN, OCN and Col-I were determined with respective human ELISA kits (R&D, USA). Optical absorbance at 450 nm was recorded spectrophotometrically and the intracellular ALP activity and contents of OPN, OCN and Col-I of osteoblasts cultured on the samples were drawn from a standard curve of absorbance vs. known standards of corresponding proteins running in parallel with the experimental samples. The results were normalized to the intracellular total protein content. Four samples for each group were tested, and each test was repeated four times (n = 4).

### Collagen secretion and ECM mineralization

Collagen secretion of osteoblast on the Ta samples was quantified by Sirius Red staining method. After 3, 7 and 14 days, the cells on the Ta samples were fixed in 4% paraformaldehyde and stained using a 0.1% solution of Sirius Red (Sigma, USA) in saturated picric acid for 18 h. After washing with 0.1 M acetic acid until red color disappeared, images of the collagen secreted by osteoblast cultured on the samples for 7 days were taken. In the quantitative analysis, the stain on the samples was then eluted with 500 mL destained solution (0.2 M NaOH/methanol 1:1) and measured using a spectrophotometer at the optical density of 540 nm. ECM mineralization of osteoblast was evaluated using Alizarin Red staining method. The samples with cells cultured for 3, 7 and 14 days were fixed in 75% ethanol for 1 h, and subsequently stained with 2% Alizarin Red (Sigma, USA) solution for 10 min. Afterwards, the samples were washed with distilled water until no color was observed. The images of ECM mineralization were taken at day 14 of culture. In the quantitative analysis, the stain was dissolved with 10% cetylpyridinium chloride in 10 mM sodium phosphate and the absorbance was monitored at 620 nm.

### Statistical analysis

The data were analyzed using SPSS 14.0 software (SPSS, USA). A one-way ANOVA followed by a Student-Newman-Keuls *post hoc* test was used to determine the level of significance. *p* < 0.05 was considered to be significant and *p* < 0.01 was considered to be highly significant.

## Conclusions

Novel NC surface layer with an average grain size of ≤20 nm has been fabricated on pure Ta by SFT technique. The higher surface energy and wettability provided by the drastically increased numbers of grain boundaries of the SFT-processed NC Ta contribute to notable increment in protein adsorption, and leads to better subsequent osteoblast cell responses. Besides, compared to CG Ta, NC Ta possesses superior corrosion resistance, which also helps to provide a preferred and stable microenvironment for cell-material interaction. Resultantly, the NC Ta surface indeed exhibits improved cytocompatibility for osteoblast, which promotes cell attachment and spreading, and shows superior viability as compared to CG Ta. Furthermore, the grain size reduction from microscale to nanoscale in Ta metal considerably accelerates the consequent proliferation, maturation and mineralization of hFOB1.19 osteoblast. Therefore, the SFT-processed pure Ta implants with the NC surface should be promising for load-bearing bone implant applications considering their combined superior mechanical property, corrosion resistance, as well as bioactivity.

## Additional Information

**How to cite this article**: Huo, W. T. *et al*. Significantly enhanced osteoblast response to nano-grained pure tantalum. *Sci. Rep.* **7**, 40868; doi: 10.1038/srep40868 (2017).

**Publisher's note:** Springer Nature remains neutral with regard to jurisdictional claims in published maps and institutional affiliations.

## Figures and Tables

**Table 1:** The corrosion potentials (*E_corr_*), corrosion current densities (*I_corr_*) obtained from PDP curves and the fitting parameters values observed in EIS of the NC and CG Ta samples tested in SBF solution.

Samples	*E*_*corr*_ (V vs. SCE)	*I*_*corr*_ (A.cm^−2^)	*R*_*s*_ (Ω)	*R*_*p*_ (10^5^ Ω.cm^2^)	*Q* (μF.cm^−2^)	*n*
NC	−0.583	1.05 ± 0.04	22.35	2.014	14.85	0.8998
CG	−0.635	1.19 ± 0.06	19.97	1.421	21.13	0.9059

**Table 2:** Primers for real time PCR.

Gene	Sequences (Forward/Reverse)	Annealing T/°C
Runx2	5′-CCTTCTGGGTTCCCGAGGT-3′/5′-AGTGGACGAGGCAAGAGTTTC-3′	60
OSX	5′-TGCGAAGCCTTGCCATACA-3′/5′-TCCTCCTGCGACTGCCCTAA-3′	60
ALP	5′-ATCTTTGGTCTGGCCCCCATG-3′/5′-ATGCAGGCTGCATACGCCAT-3′	60
OCN	5′-TCCTGAAAGCCGATGTGGT-3′/5′-AGGGCAGCGAGGTAGTGAA-3′	58
OPN	5′-ATGGCTTTCGTTGGACTTACT-3′/5′-TTTACAACAAATACCCAGATGC-3′	60
Col-I	5′-GAAGTCAGCTGCATACAC-3′/5′-AGGAAGTCCAGGCTGTCC-3′	60
GAPDH	5′-CCACCCTGTTGCTGTAGCC-3′/5′-CCCACTCCTCCACCTTTGA-3′	60

## References

[b1] Wang Q. . Tantalum implanted entangled porous titanium promotes surface osseointegration and bone ingrowth. Sci. Rep. 6, 26248 (2016). 27185196 10.1038/srep26248 PMC4869100

[b2] Findlay D. M. . The proliferation and phenotypic expression of human osteoblasts on tantalum metal. Biomaterials 25, 2215–2227 (2004). 14741587 10.1016/j.biomaterials.2003.09.005

[b3] Stiehler M. . Morphology, proliferation, and osteogenic differentiation of mesenchymal stem cells cultured on titanium, tantalum, and chromium surfaces. J. Biomed Mater. Res. Part A 86, 448–458 (2008). 10.1002/jbm.a.31602 17975813

[b4] Nie F. L., Zheng Y. F., Wang Y. & Wang J. T. Microstructures, mechanical behavior, cellular response, and hemocompatibility of bulk ultrafine-grained pure tantalum. J. Biomed. Mater. Res. B. Appl. Biomater. 102, 221–230 (2014). 23908098 10.1002/jbm.b.32998

[b5] Lu T. . Enhanced osteointegration on tantalum-implanted polyetheretherketone surface with bone-like elastic modulus. Biomaterials 51, 173–183 (2015). 25771008 10.1016/j.biomaterials.2015.02.018

[b6] Balla V. K., Banerjee S., Bose S. & Bandyopadhyay A. Direct laser processing of a tantalum coating on titanium for bone replacement structures. Acta biomaterialia 6, 2329–2334 (2010). 19931654 10.1016/j.actbio.2009.11.021 PMC2862814

[b7] Misra R. D. K. . Cellular response of preosteoblasts to nanograined/ultrafine-grained structures. Acta biomaterialia 5, 1455–1467 (2009). 19217838 10.1016/j.actbio.2008.12.017

[b8] Faghihi S., Zia S. & Taha M. F. Adipose tissue-derived stem cell response to the differently processed 316L stainless steel substrates. Tiss. Cell 44, 365–372 (2012). 10.1016/j.tice.2012.06.002 22770869

[b9] Faghihi S. . Nanostructuring of a titanium material by high-pressure torsion improves pre-osteoblast attachment. Adv. Mater. 19, 1069–1073 (2007).

[b10] Park J. W. . Enhanced osteoblast response to an equal channel angular pressing-processed pure titanium substrate with microrough surface topography. Acta biomaterialia 5, 3272–3280 (2009). 19426841 10.1016/j.actbio.2009.04.038

[b11] Huang R., Lu S. & Han Y. Role of grain size in the regulation of osteoblast response to Ti-25Nb-3Mo-3Zr-2Sn alloy. Colloids surf. B. Biointerfaces 111, 232–241 (2013). 23831591 10.1016/j.colsurfb.2013.06.007

[b12] Estrin Y. . Accelerated stem cell attachment to ultrafine grained titanium. Acta biomaterialia 7, 900–906 (2011). 20887818 10.1016/j.actbio.2010.09.033

[b13] Xie K. Y. . Nanocrystalline beta-Ti alloy with high hardness, low Young’s modulus and excellent *in vitro* biocompatibility for biomedical applications. Mater. Sci. Eng. C 33, 3530–3536 (2013). 10.1016/j.msec.2013.04.044 23706243

[b14] Saldana L. . *In vitro* biocompatibility of an ultrafine grained zirconium. Biomaterials 28, 4343–4354 (2007). 17624424 10.1016/j.biomaterials.2007.06.015

[b15] Liu X. C., Zhang H. W. & Lu K. Strain-induced ultrahard and ultrastable nanolaminated structure in nickel. Science 342, 337–340 (2013). 24136963 10.1126/science.1242578

[b16] Liu X. C., Zhang H. W. & Lu K. Formation of nano-laminated structure in nickel by means of surface mechanical grinding treatment. Acta Mater. 96, 24–36 (2015).

[b17] Zhang Y. S. . Formation of nanocrystalline structure in tantalum by sliding friction treatment. Int. J Ref. Met. Hard. Mater. 45, 71–75 (2014).

[b18] Zhang Y. S. . Deformation twinning and localized amorphization in nanocrystalline tantalum induced by sliding friction. Mater. Lett. 127, 4–7 (2014).

[b19] Zhang Y. S. . Surface nanocrystallization of Cu and Ta by sliding friction. Mater. Sci. Eng. A 607, 351–355 (2014).

[b20] Cheng Y.-T. & Rodak D. E. Is the lotus leaf superhydrophobic? Appl. Phys. Lett. 86, 144101 (2005).

[b21] Martines E. . Superhydrophobicity and superhydrophilicity of regular nanopatterns. Nano. Lett. 5, 2097–2103 (2005). 16218745 10.1021/nl051435t

[b22] Anselme K. . The interaction of cells and bacteria with surfaces structured at the nanometre scale. Acta biomaterialia 6, 3824–3846 (2010). 20371386 10.1016/j.actbio.2010.04.001

[b23] Faghihi S. . Cellular and molecular interactions between MC3T3-E1 pre-osteoblasts and nanostructured titanium produced by high-pressure torsion. Biomaterials 28, 3887–3895 (2007). 17568665 10.1016/j.biomaterials.2007.05.010

[b24] Huang R. & Han Y. The effect of SMAT-induced grain refinement and dislocations on the corrosion behavior of Ti-25Nb-3Mo-3Zr-2Sn alloy. Mater. Sci. Eng. C 33, 2353–2359 (2013). 10.1016/j.msec.2013.01.068 23498269

[b25] Li H. F., Zhou F. Y., Li L. & Zheng Y. F. Design and development of novel MRI compatible zirconium- ruthenium alloys with ultralow magnetic susceptibility. Sci. Rep. 6, 24414 (2016). 27090955 10.1038/srep24414 PMC4836298

[b26] Orlov D., Ralston K. D., Birbilis N. & Estrin Y. Enhanced corrosion resistance of Mg alloy ZK60 after processing by integrated extrusion and equal channel angular pressing. Acta Mater. 59, 6176–6186 (2011).

[b27] Seong J. W. & Kim W. J. Development of biodegradable Mg-Ca alloy sheets with enhanced strength and corrosion properties through the refinement and uniform dispersion of the Mg_2_Ca phase by high-ratio differential speed rolling. Acta biomaterialia 11, 531–542 (2015). 25246310 10.1016/j.actbio.2014.09.029

[b28] Xu W. . A high-specific-strength and corrosion-resistant magnesium alloy. Nat. Mater. 14, 1229–1235 (2015). 26480229 10.1038/nmat4435

[b29] Jiang P. . Electrochemical construction of micro–nano spongelike structure on titanium substrate for enhancing corrosion resistance and bioactivity. Electrochim Acta 107, 16–25 (2013).

[b30] Gnedenkov A. S., Sinebryukhov S. L., Mashtalyar D. V. & Gnedenkov S. V. Protective properties of inhibitor-containing composite coatings on a Mg alloy. Corros. Sci. 102, 348–354 (2016).

[b31] Vinogradov A., Mimaki T., Hashimoto S. & Valiev R. On the corrosion behaviour of ultra-fine grain copper. Scr. Mater. 41, 319–326 (1999).

[b32] Balyanov A. Corrosion resistance of ultra fine-grained Ti. Scr. Mater. 51, 225–229 (2004).

[b33] Nie F. L., Zheng Y. F., Wei S. C., Hu C. & Yang G. *In vitro* corrosion, cytotoxicity and hemocompatibility of bulk nanocrystalline pure iron. Biomed. Mater. 5, 065015 (2010). 21079282 10.1088/1748-6041/5/6/065015

[b34] Nie F. L. & Zheng Y. F. Surface chemistry of bulk nanocrystalline pure iron and electrochemistry study in gas-flow physiological saline. J. Biomed. Mater. Res. B. Appl. Biomater. 100, 1404–1410 (2012). 22566153 10.1002/jbm.b.32713

[b35] Faghihi S., Li D. & Szpunar J. A. Tribocorrosion behaviour of nanostructured titanium substrates processed by high-pressure torsion. Nanotechnology 21, 485703 (2010). 21063052 10.1088/0957-4484/21/48/485703

[b36] Zhao Y. . Improved corrosion resistance and cytocompatibility of magnesium alloy by two-stage cooling in thermal treatment. Corros. Sci. 59, 360–365 (2012).

[b37] Liu X. . Influence of substratum surface chemistry/energy and topography on the human fetal osteoblastic cell line hFOB 1.19: Phenotypic and genotypic responses observed *in vitro*. Biomaterials 28, 4535–4550 (2007). 17644175 10.1016/j.biomaterials.2007.06.016 PMC2705827

[b38] Zhou J. & Zhao L. Multifunction Sr, Co and F co-doped microporous coating on titanium of antibacterial, angiogenic and osteogenic activities. Sci. Rep. 6, 29069 (2016). 27353337 10.1038/srep29069 PMC4926257

[b39] Zhao L. . Mechanism of cell repellence on quasi-aligned nanowire arrays on Ti alloy. Biomaterials 31, 8341–8349 (2010). 20667412 10.1016/j.biomaterials.2010.07.036

[b40] Zhou J., Li B., Han Y. & Zhao L. The osteogenic capacity of biomimetic hierarchical micropore/nanorod-patterned Sr-HA coatings with different interrod spacings. Nanomedicine 12, 1161–1173 (2016). 26961465 10.1016/j.nano.2016.01.011

[b41] Nie F. L. . *In vitro* and *in vivo* studies on nanocrystalline Ti fabricated by equal channel angular pressing with microcrystalline CP Ti as control. J. Biomed. Mater. Res. Part A 101, 1694–1707 (2013). 10.1002/jbm.a.34472 23184756

[b42] Huang R., Zhuang H. & Han Y. Second-phase-dependent grain refinement in Ti-25Nb-3Mo-3Zr-2Sn alloy and its enhanced osteoblast response. Mater. Sci. Eng. C 35, 144–152 (2014). 10.1016/j.msec.2013.10.037 24411362

[b43] Bagherifard S. . The influence of nanostructured features on bacterial adhesion and bone cell functions on severely shot peened 316L stainless steel. Biomaterials 73, 185–197 (2015). 26410786 10.1016/j.biomaterials.2015.09.019

[b44] Wang W. . The role of the Wnt/β-catenin pathway in the effect of implant topography on MG63 differentiation. Biomaterials 33, 7993–8002 (2012). 22889483 10.1016/j.biomaterials.2012.07.064

[b45] Nakashima K. . The novel zinc finger-containing transcription factor osterix is required for osteoblast differentiation and bone formation. Cell 108, 17–29 (2002). 11792318 10.1016/s0092-8674(01)00622-5

[b46] Komori T. . Targeted disruption of Cbfa1 results in a complete lack of bone formation owing to maturational arrest of osteoblasts. Cell 89, 755–764 (1997). 9182763 10.1016/s0092-8674(00)80258-5

[b47] Zhao L. . The osteogenic activity of strontium loaded titania nanotube arrays on titanium substrates. Biomaterials 34, 19–29 (2013). 23046755 10.1016/j.biomaterials.2012.09.041

[b48] Liu C. . Biodegradable Mg-Cu alloys with enhanced osteogenesis, angiogenesis, and long-lasting antibacterial effects. Sci. Rep. 6, 27374 (2016). 27271057 10.1038/srep27374 PMC4895436

[b49] Zhou J., Li B., Lu S., Zhang L. & Han Y. Regulation of osteoblast proliferation and differentiation by interrod spacing of Sr-HA nanorods on microporous titania coatings. ACS. Appl. Mater. Interfaces 5, 5358–5365 (2013). 23668394 10.1021/am401339n

[b50] Ha S. W., Jang H. L., Nam K. T. & Beck G. R. Jr. Nano-hydroxyapatite modulates osteoblast lineage commitment by stimulation of DNA methylation and regulation of gene expression. Biomaterials 65, 32–42 (2015). 26141836 10.1016/j.biomaterials.2015.06.039 PMC4508253

[b51] Saffarian Tousi N. . Combinatorial effect of Si^4+^, Ca^2+^, and Mg^2+^ released from bioactive glasses on osteoblast osteocalcin expression and biomineralization. Mater. Sci. Eng. C 33, 2757–2765 (2013). 10.1016/j.msec.2013.02.044 23623093

[b52] Kokubo T. & Takadama H. How useful is SBF in predicting *in vivo* bone bioactivity? Biomaterials 27, 2907–2915 (2006). 16448693 10.1016/j.biomaterials.2006.01.017

